# Epidemiology of tularemia in the countries of the WHO Eastern Mediterranean Region (EMRO): A systematic review and meta-analysis

**DOI:** 10.1371/journal.pntd.0012141

**Published:** 2024-05-10

**Authors:** Mohammad Sholeh, Safoura Moradkasani, Saber Esmaeili

**Affiliations:** 1 Department of Bacteriology, Pasteur Institute of Iran, Tehran, Iran; 2 Department of Epidemiology and Biostatistics, Research Center for Emerging and Reemerging Infectious Diseases, Pasteur Institute of Iran, Tehran, Iran; 3 National Reference Laboratory for Plague, Tularemia and Q Fever, Research Centre for Emerging and Reemerging Infectious Diseases, Pasteur Institute of Iran, Akanlu, KabudarAhang, Hamadan, Iran; USAMRIID: US Army Medical Research Institute of Infectious Diseases, UNITED STATES

## Abstract

**Background:**

*Francisella tularensis*, the bacterium that causes tularemia, has been a persistent and widespread pathogen in various regions of the world for centuries. *Francisella tularensis* can affect humans and various domestic and wild animals. The current study aimed to determine the epidemiological status of tularemia in countries of the WHO Eastern Mediterranean Region (EMRO) through a systematic review and meta-analysis.

**Methods:**

All included studies were identified through a systematic search of online databases, including Scopus, PubMed, Web of Science, and EMBASE, through July 26, 2022, using keywords and suitable combinations. We focused on cross-sectional studies investigating the prevalence of *F*. *tularensis*. The weighted pooled prevalence was calculated using a random-effects model.

**Results:**

A total of 206 studies were identified, of which 20 were finally included in the analysis. The human seroprevalence of tularemia in WHO-EMRO countries was 6.2% (95% CI, 4.2 9.2). In the subgroup analysis, anti-*F*. *tularensis* antibodies were found in 6.92% and 5.5% of the high-risk individuals and Iran, respectively. The pooled prevalence of *F*. *tularensis* in environmental samples (water and soil) from the WHO-EMRO countries was 5.8% (9.4% by PCR and 0.5% by culture). In addition, 2.5% (95% CI, 0.2 0.22.7) of ticks in WHO-EMRO countries were positive for *F*. *tularensis*. The pooled prevalence of *F*. *tularensis* in rodents is 2.0% (1.1% by PCR and 3.7% by serology). In addition, 0.6% of domestic ruminants (0.4% by PCR and 2.4% by serology) were positive for *F*. *tularensis* in WHO-EMRO countries.

**Conclusion:**

According to the results of the present study, tularemia is an endemic but neglected disease in the WHO-EMRO region. However, most studies on tularemia are limited to a few countries in this region. Studies on tularemia in human populations, reservoirs, and vectors have been conducted in all countries in the WHO-EMRO region to obtain more detailed information about the epidemiology of tularemia in these regions.

## Author summary

Tularemia is a bacterial zoonotic disease caused by *Francisella tularensis*. This bacterium can infect animals (domestic and wild vertebrates), invertebrates, and humans. *Francisella tularensis* is transferred to humans through contact with infected wild animals (, inhalation of infected aerosols, arthropod bites, consumption of contaminated water or contaminated food, and swimming and contact in polluted hydro-telluric environments. The mortality rate associated with this disease ranges from 2 to 60% in the absence of proper antibiotic treatment. The prevalence of *F*. *tularensis* varies significantly between countries, as these pathogens can infect a wide range of hosts and have multiple vectors. However, there needs to be complete data on the geographical distribution of different species of *F*. *tularensis*, as well as the status of reservoirs, vectors, and human cases in most parts of the world. Therefore, this study aimed to determine *F*. *tularensis* prevalence in the countries of the WHO Eastern Mediterranean Region (WHO-EMRO). Based on our study, the human seroprevalence of tularemia in WHO-EMRO countries was 6.2%. The pooled prevalence of *F*. *tularensis* in environmental samples (water and soil), ticks, rodents, and domestic ruminants were 5.8%, 2.5%, 2.0%, and 0.6% in the WHO-EMRO countries, respectively.

## Introduction

Tularemia is a bacterial zoonotic disease caused by *Francisella tularensis*. This bacterium can infect animals (domestic and wild vertebrates), invertebrates, and humans. *Francisella tularensis* has three subspecies: *tularensis* (type A), *holarctica* (type B), *mediasiatica* (has never been isolated from human cases), and *Francisella novicida*, while officially classified as a distinct species, but frequently regarded as a de facto fourth subspecies [1]. Type A is highly virulent and causes most human tularemia cases in North America; in contrast, type B is found in the entire Northern Hemisphere, and all cases of tularemia belong to this type in Asia and Europe. *Francisella tularensis* is transferred to humans through contact with infected wild animals (especially hares and small rodents), inhalation of infected aerosols, arthropod bites (mainly Ixodidae ticks and mosquitoes), consumption of contaminated water or contaminated food, and swimming and contact in polluted hydro-telluric environments [2]. After a short incubation period (3–5 days, maximum two weeks), infected individuals displayed flu-like symptoms. Depending on the entry route of *F*. *tularensis* into the body, tularemia can progress into six different clinical forms in humans [3].

These include typhoidal (severe sepsis with confusion), oculoglandular (conjunctivitis with cervical or pre-tracheal lymphadenopathy), oropharyngeal (pharyngitis with cervical lymphadenopathy), and ulceroglandular and glandular forms (regional lymphadenopathy with or without a skin inoculation lesion). Meningitis, meningoencephalitis, heart, bone, soft tissue infections, and lymphadenopathy are complications of tularemia. People with tularemia lymphadenopathy progress to lymph node suppuration in approximately 30% of cases [4–6]. The mortality rate associated with type A infections ranges from 5 to 15% in the absence of antibiotic treatment, increasing to 30 to 60% for cases involving severe pneumonia or septicemia. However, timely antibiotic intervention can effectively reduce the fatality rate to less than 2% [7].

The diagnosis of tularemia can frequently be delayed due to the late consultation of patients with mild symptoms and the lack of specificity of clinical signs [8]. *Francisella tularensis* isolation from clinical samples is often challenging, with a success rate of less than 10% [8,9]. When exudate or tissue samples are available, PCR-based approaches can be used to identify localized tularemia. Early stages of tularemia, such as acute pneumonia, oculoglandular, or oropharyngeal forms, or late stages, such as testing surgically excised suppurated lymph nodes, can be detected by PCR tests. Given diagnostic tests for tularemia (especially culture tests and PCR tests) are not routinely available in many EMRO countries, so serological tests are considered the primary method for tularemia diagnosis [2,9,10].

In most countries, tularemia (except in some countries, such as the USA, France, Spain, Sweden, and Turkey), is a neglected disease because the diagnosis requires specific laboratory diagnostic tests. This lack of diagnosis translates to a dearth of up-to-date data on *F*. *tularensis* in most nations. The prevalence of *F*. *tularensis* varies significantly between countries, as these pathogens can infect a wide range of hosts and have multiple vectors. However, there needs to be complete data on the geographical distribution of different species of *F*. *tularensis*, as well as the status of reservoirs, vectors, and human cases in most parts of the world. Therefore, this study aimed to determine *F*. *tularensis* prevalence in the countries of the WHO Eastern Mediterranean Region (WHO-EMRO).

## Methods

### Eligibility criteria

This meta-analysis included studies that reported the proportion of positive tests, specified the sample size, and had full-text English versions available. Any language other than English, case reports, single-arm, cohort, and pharmacokinetic studies were excluded.

### Search strategy

All included studies were identified through a systematic search of online databases, including Scopus, PubMed, Web of Science, and EMBASE, until July 26, 2022. For other databases, search syntax was adapted according to the following: (“*Francisella tularensis*” OR “*F*. *tularensis*” OR Tularemia OR “rabbit fever” OR “deer fly fever”) AND (Afghanistan OR Bahrain OR Djibouti OR Egypt OR Iran OR Iraq OR Jordan OR Kuwait OR Lebanon OR Libya OR Morocco OR Palestinian OR Oman OR Pakistan OR Palestine OR Qatar OR “Saudi Arabia” OR Somalia OR Sudan OR Syrian OR Tunisia OR “United Arab Emirates” OR Yemen).

### Selection process

After importing and eliminating duplicates from the outcomes of a thorough online database search using EndNote (version 20), two individuals (SMK and MS) conducted separate searches and analyses of pertinent papers to eliminate bias. Any discrepancies were examined by a third author (SE), who concluded.

### Data collection process

#### Data items

The first author(s), publication year, nation, diagnostic technique, sample source, number of positive tests, and total number of subjects (sample size) were among the information extracted. Two authors (S-MK and N-S) separately extracted the essential data to prevent any errors in data extraction and understood the discrepant data.

#### Study risk of bias assessment

The JBI checklist was employed to assess the quality of the included papers, owing to the inclusion of cross-sectional research. Two authors (SMK and MS) completed the quality assessment separately. The third author (SE) examined any discrepancies and made judgments.

#### Synthesis methods

The main objective of this study was to determine the seroprevalence of *F*. *tularensis* in human samples from the WHO-EMRO Region. The pooled seroprevalence was computed using the number of positive tests and the entire population (sample size). Estimating *F*. *tularensis* molecular prevalence in environmental samples (Environmental water or soil) represented the second outcome.

The third outcome was estimating the molecular prevalence of *F*. *tularensis* among arthropods. The fourth outcome was the estimation of the molecular and seroprevalence of *F*. *tularensis* in rodents. The fifth outcome estimates the seroprevalence and molecular prevalence of *F*. *tularensis* in ruminants.

#### Statistics

The percentage was used as the outcome measure in the analysis. A random effects model was used to fit the data. The DerSimonian-Laird estimator was used to calculate the measure of heterogeneity [11]. In addition, the Q-test for heterogeneity and **I**^**2**^ statistics were performed. A prediction interval for the true outcomes is also provided if heterogeneity is detected (i.e., τ2>0, regardless of the Q-test results) [12]. Meta-regression analysis was conducted to investigate the differences in prevalence between countries for people with high-risk and low-risk jobs based on publication year, quality assessment score, and subgroup analysis.

Studentized residuals and Cook’s distances were used to examine whether studies were outliers and/or influential in the model context [13]. Studies with a studentized residual larger than the 100×(1–0.05/(2×k)) th percentile of a standard normal distribution were considered potential outliers (i.e., using a Bonferroni correction with two-sided α = 0.05, for k studies included in the meta-analysis). Cook’s distances greater than the median and six times the interquartile range are considered influential studies. Rank correlation and regression tests using the standard error of the observed outcomes as predictors were used to assess funnel plot asymmetry [14,15]. The analysis was conducted using the R software (version 4.2.1) and the metafor package (version 3.8.1).

## Results

### Descriptive statistics

**Fig 1:**
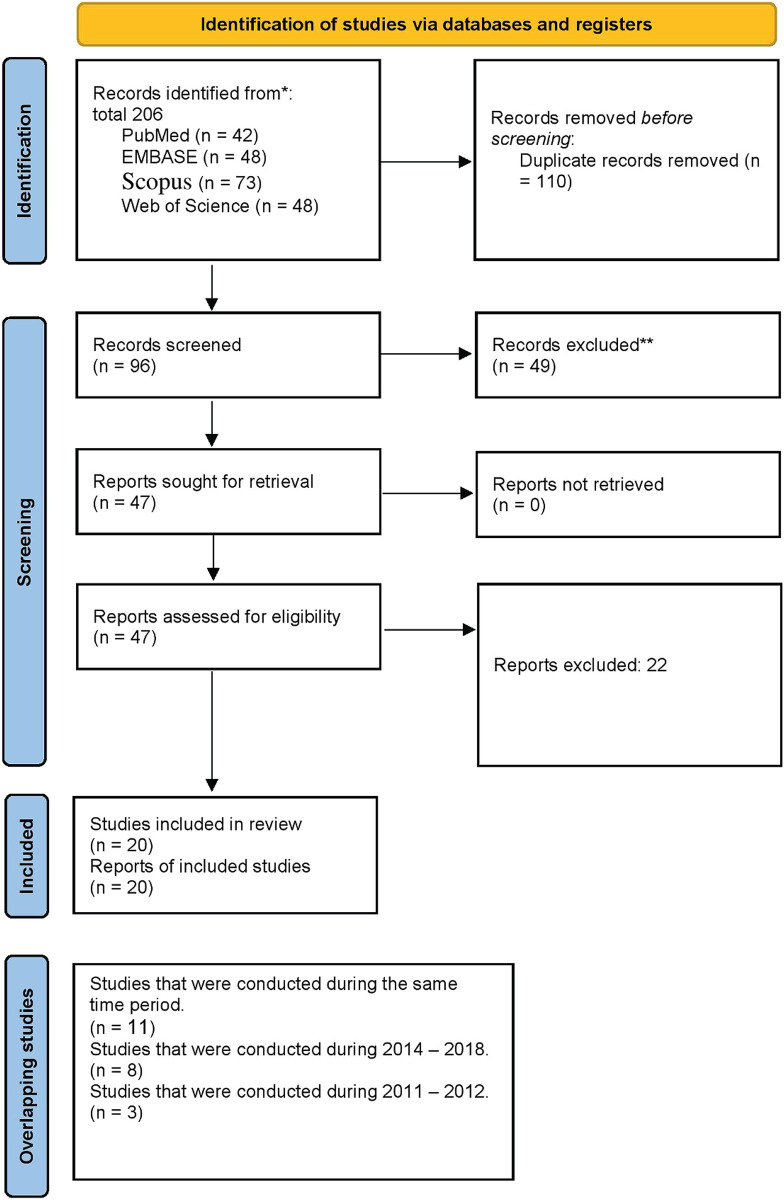
PRISMA flow diagram of the included articles. The process of selecting studies for inclusion in this systematic review or meta-analysis.

**Fig 2:**
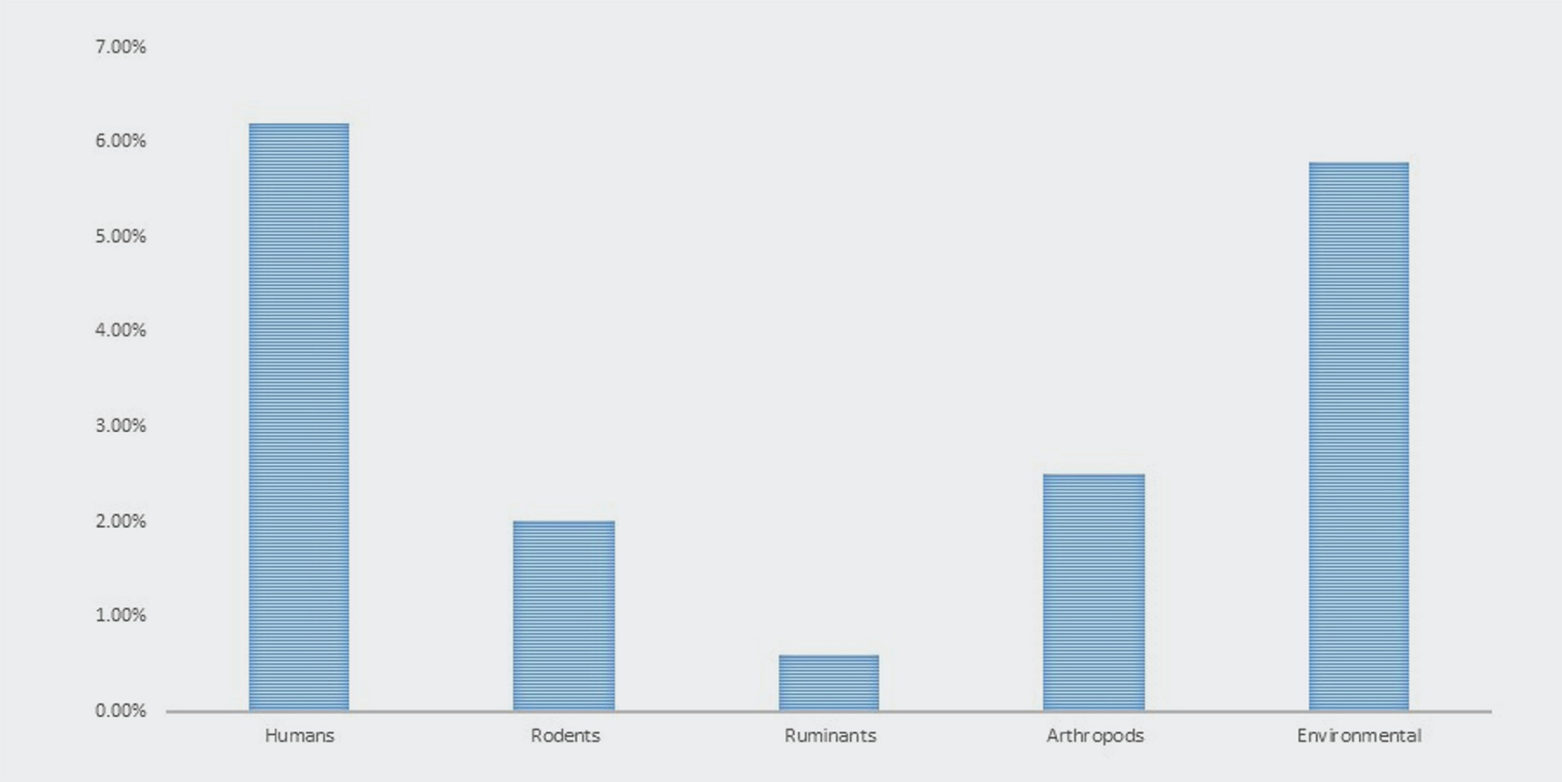
The prevalence of *F*. *tularensis* in each of the sample populations (humans, rodents, ruminants, arthropods, environmental).

The systematic search yielded 206 records using the reference manager software (EndNote version 20), and 110 duplicate articles were removed. The title abstracts of the 96 articles were reviewed, 47 full-text articles were evaluated, and 27 articles were excluded. Eventually, this systematic review and meta-analysis included 20 eligible studies (Table 1) [16–35]. The PRISMA flowchart summarizes the screening and selection processes (Fig 1). There have been five reports from different nations: Egypt, Iran, Jordan, Pakistan, and the United Arab Emirates. The majority of reports were from Iran (number of reports from Iran = 16, 72.72%). The period from 1973 to 2022 is covered in these reports. Over eight years, from 2011 to 2018, 11 research investigations with overlapping timeframes were conducted in Iran. Eight investigations were conducted between 2014 and 2018, and three investigations were conducted between 2011 and 2012. Studies have been conducted on human and environmental samples, including soil and water sources, ruminants, and rodents. *Francisella tularensis* was diagnosed using three different diagnostic methods including culture, PCR, and serology. The prevalence of *F*. *tularensis* in each of the sample populations (humans, rodents, ruminants, arthropods, environmental) was shown in Fig 2.

**Table 1:** Included articles for this study.

Author (year)	Enrolment period	Country	Quality score	Reference
**Aghamohammad. et all (2022)**	2022–2022	Iran	5	[17]
**E. Mostafavi.et al (2018)**	2014–2015	Iran	5	[16]
**Shabbir, M. Z.et al (2015)**	2015–2015	Pakistan	5	[18]
**Pourhossein, B.et al (2015)**	2013–2013	Iran	5	[19]
**Nighat Perveen.et al (2021)**	2019–2020	UAE	5	[20]
**Majid Hemati. et al (2019)**	2014–2017	Iran	6	[21]
**Arata, A.et al (1973)**	1969–1970	Iran	6	[22]
**Khoshdel, A.et al (2014)**	2011–2011	Iran	6	[23]
**Rohani, M.et al (2019)**	2015–2015	Iran	6	[24]
**Rahravani, M.et al (2018)**	2018–2018	Iran	6	[25]
**Mostafavi, E. Et al (2017)**	2017–2017	Iran	6	[26]
**Will K. Reeves. Et al (2006)**	2006–2006	Egypt	6	[27]
**Saber Esmaeili.et al (2019)**	2017–2017	Iran	7	[28]
**Saber Esmaeili.et al (2014)**	2011–2011	Iran	7	[29]
**Saber Esmaeili.et al (2019)**	2015–2015	Iran	7	[30]
**Saber Esmaeili.et al (2013)**	2011–2012	Iran	7	[31]
**Muhammad, J.et al (2019)**	2011–2015	Pakistan	7	[32]
**Nahed H. Ghoneim. et al. (2017)**	2017–2017	Egypt	8	[33]
**Mohammad M. Obaidat.et al (2020)**	2015–2016	Jordan	8	[34]
**Ahangari Cohan.et al (2020)**	2018–2018	Iran	9	[35]

### Seroprevalence of tularemia in Human

**Fig 3:**
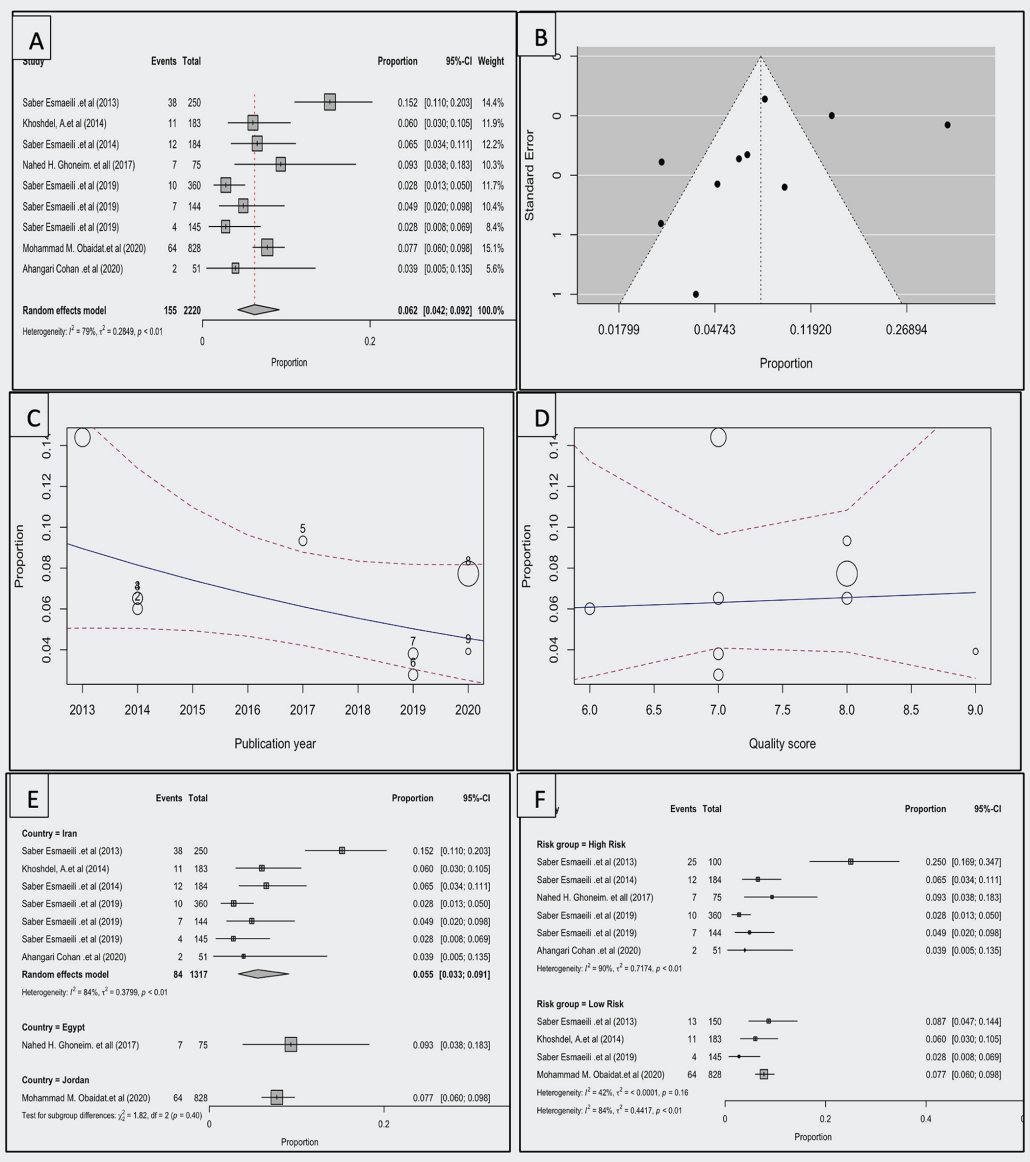
A Forest plot showing the observed human *F*. *tularensis* seroprevalence and the estimate of the random-effects model, B; Funnel plot for a meta-analysis of studies of the human *F*. *tularensis* seroprevalence, C; Scatterplot of observed human *F*. *tularensis* seroprevalence over years, D; Scatterplot of observed human *F*. *tularensis* seroprevalence across the NOS checklist quality score, E; Forest plot showing the observed human *F*. *tularensis* seroprevalence in countries and the estimate of the random-effects model, F; Forest plot showing the observed human *F*. *tularensis* seroprevalence in a different risk group and the estimate of the random-effects model.

Nine studies were included in this analysis [23,28,29,31,33–36]. Based on the random-effects model, the seroprevalence of tularemia in at-risk groups of humans including laboratory employees, farmers, ranchers, hunters, veterinarians, nature conservation officers, butchers, and slaughterhouse workers was 0.062 (95% CI, 0.042–0.092). The average proportion differed significantly from zero (z = -13.989, *p<*0.001). In Fig 3A, a forest plot of the seroprevalence of tularemia in humans can be seen, along with an estimate based on a random-effects model. According to the Q test, the true outcomes were heterogeneous (Q (9) = 55.631, *p<*0.001, τ^2^ = 0.431, **I**^**2**^ = 83.822%). The funnel plot of the estimates is shown in Fig 3B. Both rank correlation and regression tests indicated no asymmetry in the funnel plot (*p =* 0.108 and *p =* 0.167, respectively).

The studentized residuals indicated no outliers in this model; therefore, there were no indications of outliers. According to Cook’s distance, none of the studies could be considered overly influential.

Based on the moderator analysis, there was no significant change in prevalence trends over time (correlation = -0.103 (95% CI, [-0.241, 0.035], *p =* 0.143). The prevalence and quality score were not significantly correlated (coefficient = 0.040 [95% confidence interval, -0.514, 0.593], *p =* 0.888) (Fig 3D). Differences between nations were not significantly different (*p =* 0.065) (Fig 3E). Risk groups did not differ significantly (*p =* 0.400) (Fig 3F).

### Prevalence of *F*. *tularensis* in environmental samples

**Fig 4:**
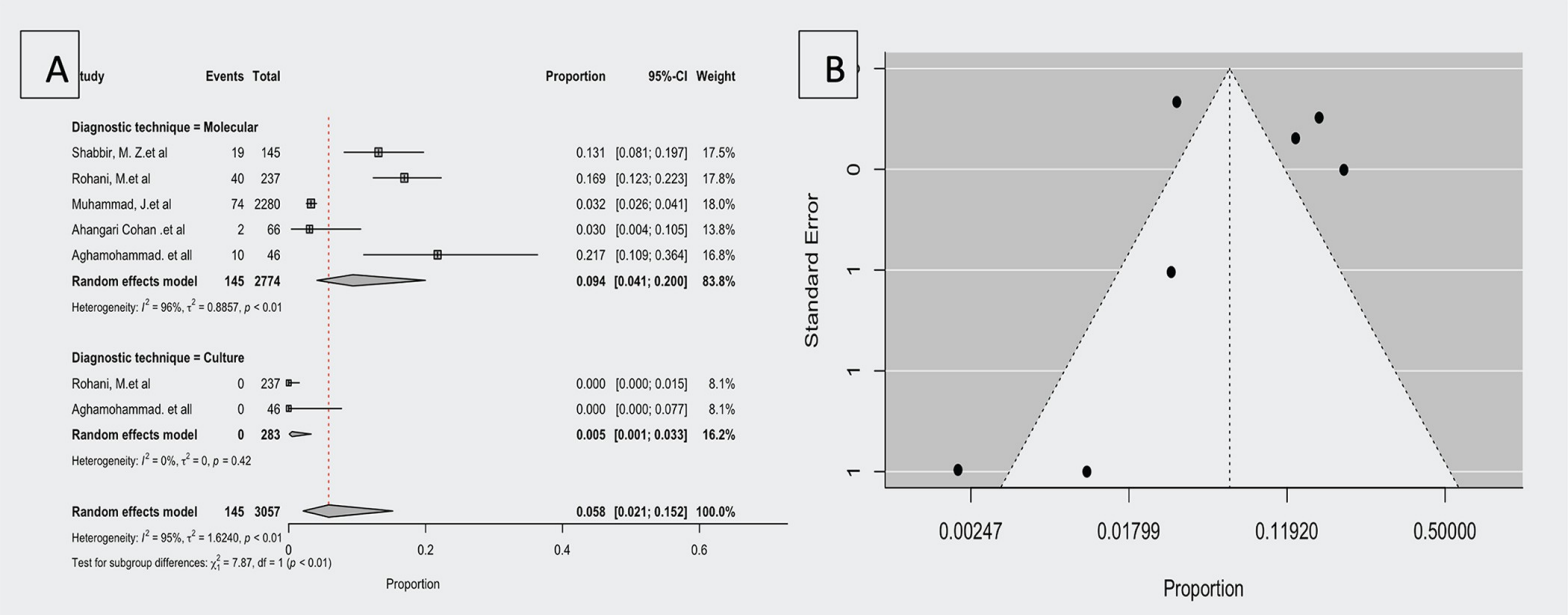
A; Forest plot showing the observed prevalence in environmental samples in different diagnostic techniques and the estimate of the random-effects model, B; Funnel plot for a meta-analysis of studies of the *F*. *tularensis* prevalence in environmental samples.

The prevalence of *F*. *tularensis* was significant (*p<*0.005) in the environmental samples in the subgroup analysis based on different diagnostic techniques. According to five studies that investigated the prevalence of *F*. *tularensis* in soil and surface water samples using molecular techniques [17,18,32,35,37], the prevalence ranged from 3 to 21.7%. The estimated average proportion based on the random-effects model was μ = 0.094 (95% CI,0.041–0.200). Therefore, the average outcome differed significantly from zero (z = -4.402, *p<*0.001). A forest plot showing the observed prevalence in environmental samples using different diagnostic techniques and the estimate of the random effects model is shown in Fig 4A. According to the Q test, the true outcomes appeared to be heterogeneous (Q (4) = 102.140, *p<*0.001, τ2 = 1.211, I^2^ = 96.084%) (Fig 3A). There is an outlier in this model based on studentized residuals, which show that one study [3] has a value greater than two. Cook’s distances indicated that one study [3] is overly influential. Consequently, when this study is removed, the overall proportion is 0.142 (95% CI, 0.091–0.215). The funnel plot of the estimates is shown in Fig 4B. No funnel plot asymmetry was based on rank correlation or regression analysis (*p =* 1.000 and *p =* 0.665, respectively). According to two studies investigating the prevalence of *F*. *tularensis* in environmental samples by culture techniques **(17, 37)**, the observed proportion was 0.000. Based on the random-effects model, the average proportion was μ = 0.005 (95% CI,0.001–0.033). According to the Q-test, the true outcomes were homogenous (Q(1) = 0.661, *p<*0.416, τ2 = 0.000, I^2^ = 0.00%).

### Prevalence of *F*. *tularensis* in arthropods

**Fig 5:**
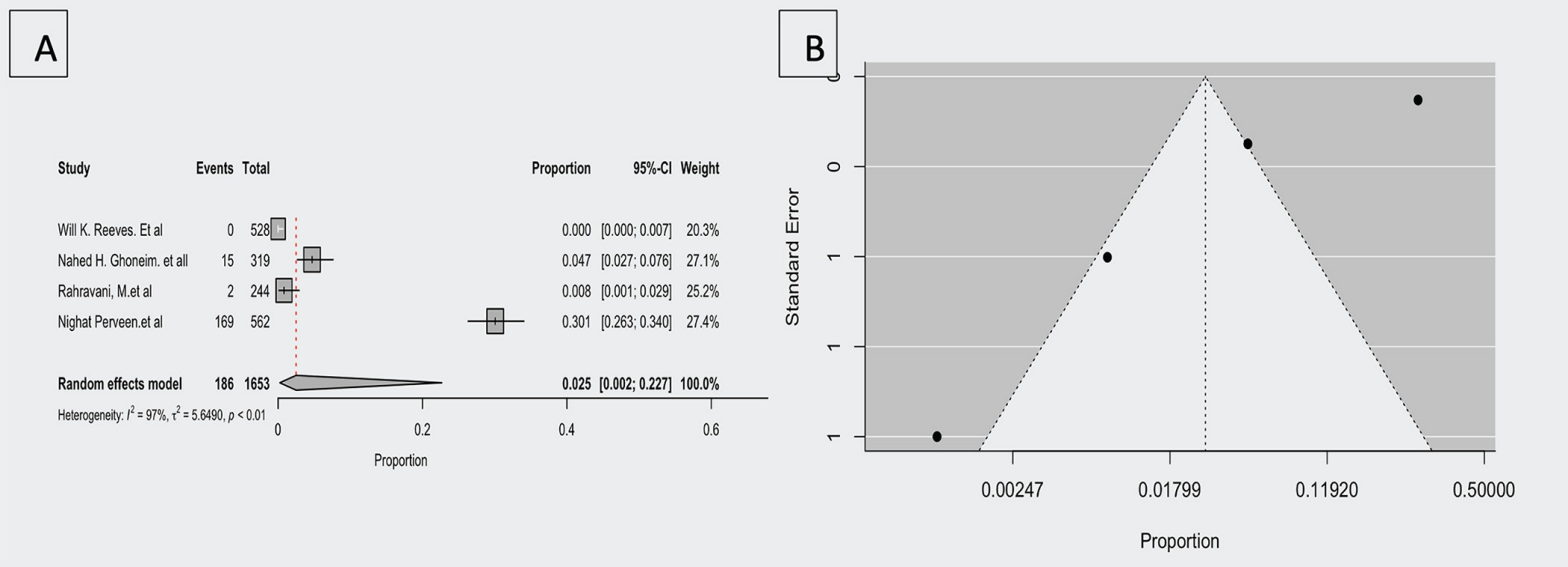
A; Forest plot showing the observed *F*. *tularensis* molecular prevalence in arthropods and the estimate of the random-effects model, B; Funnel plot for a meta-analysis of studies of the arthropods *F*. *tularensis* molecular prevalence.

According to four studies [20,25,27,33], the molecular prevalence of *F*. *tularensis* in arthropods ranges from 0.000 to 0.301. The estimated average proportion based on the random-effects model was μ = 0.025 (95% CI,0.002–0.227). Therefore, the average prevalence differed significantly from zero (z = -3.605, *p<*0.001). A forest plot showing the observed prevalence and the estimate based on the random effects model is shown in Fig 5A.

According to the Q test, the true outcomes appeared to be heterogeneous (Q (3) = 103.716, *p<*0.001, τ^2^ = 3.366, I^2^ = 97.107%). This model had no outliers based on the studentized residuals, as none of the studies exceeded 2.498; therefore, this model did not indicate outliers. According to Cook’s distance, none of the studies could be considered overly influential. There is no evidence of funnel plot asymmetry (*p<*0.001) from the regression test, but rank correlation does not indicate any such asymmetry (*p =* 1.000).

### Prevalence of *F*. *tularensis* in rodents

According to four studies (three seroprevalence, three molecular prevalence, and one culture technique positive proportion has been reported) that have investigated the prevalence of *F*. *tularensis* in rodents [20,25,27,33], the observed proportion ranged from 0.000 to 0.111. The estimated average proportion based on the random-effects model was μ = 0.058 (95% CI,0.010–0.039). Therefore, the average outcome differed significantly from zero (z = -10.587, *p<*0.001).

According to the Q test, the true outcomes appeared heterogeneous (Q(6) = 14.357, *p =* 0.026, τ^2^ = 0.462, I^2^ = 58.208%).

Due to the significance of heterogeneity subgroup analysis was performed, the difference between groups was significant (*p =* 0.05), hence based on the diagnosis technique, seroprevalence was μ = 0.037 (95% CI, 0.016 to 0.085), the molecular prevalence was μ = 0.011 (95% CI, 0.005 to 0.023), and the proportion of culture positive was μ = 0.020 (95% CI, 0.010 to 0.039).

The studentized residuals showed that none of the studies had a value greater than 2.699, indicating that the model had no outliers. According to Cook’s distances, one study [2] could be considered overly influential, and by removing this study, the overall proportion was 0.159 (95% CI, 0.009–0.025).

**Fig 6:**
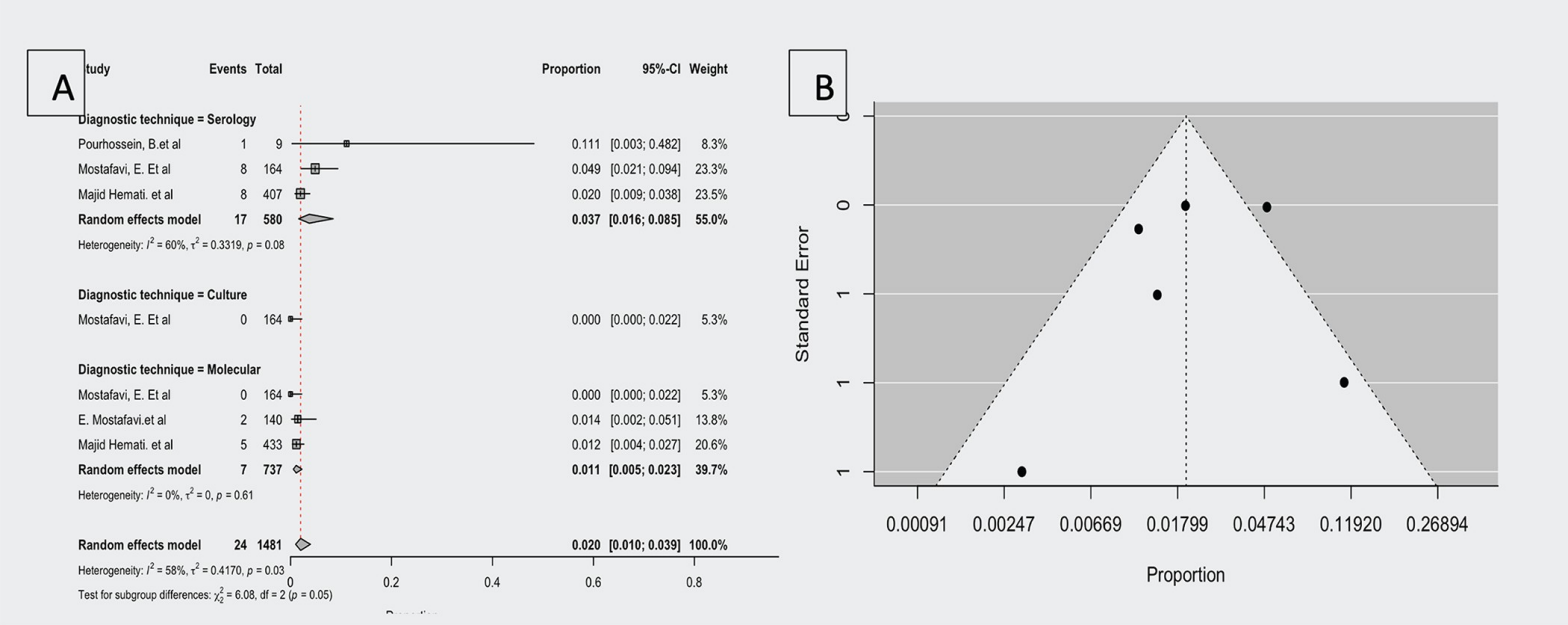
Forest plot showing the observed *F*. *tularensis* prevalence in rodents and the estimate of the random-effects model, B: Funnel plot for a meta-analysis of studies of the *F*. *tularensis* prevalence in rodents.

Fig 6B shows the funnel plot of the estimates. There was no evidence of funnel plot asymmetry in the rank correlation or regression test (*p =* 0.357 and *p =* 0.291, respectively).

Significant differences existed between the diagnosis techniques subgroups (Q (2) = 6.08, *p =* 0.048). Compared with molecular and culture diagenetic techniques, serology diagenetic has a higher proportion of reports (0.37; 95% CI, 0.016–0.085) than molecular diagnosis (0.011; 95% CI, 0.005–0.023).

### Prevalence of *F*. *tularensis* in ruminants

According to five (three seroprevalences, three molecular prevalence’s, and one culture-positive prevalence have been reported) studies investigating the prevalence of *F*. *tularensis* in ruminants [20,25,27,33], the observed proportion ranged from 0.000 to 0.062. Based on the random-effects model, the estimated average proportion was μ = 0.006 (95% CI,0.001–0.033). Therefore, the average outcome differed significantly from zero (z = -8.323, *p<*0.001).

According to the Q-test, the true outcomes were heterogeneous (Q (7) = 42.535, *p<*0.001, τ^2^ = 1.566, I^2^ = 83.543%).

**Fig 7:**
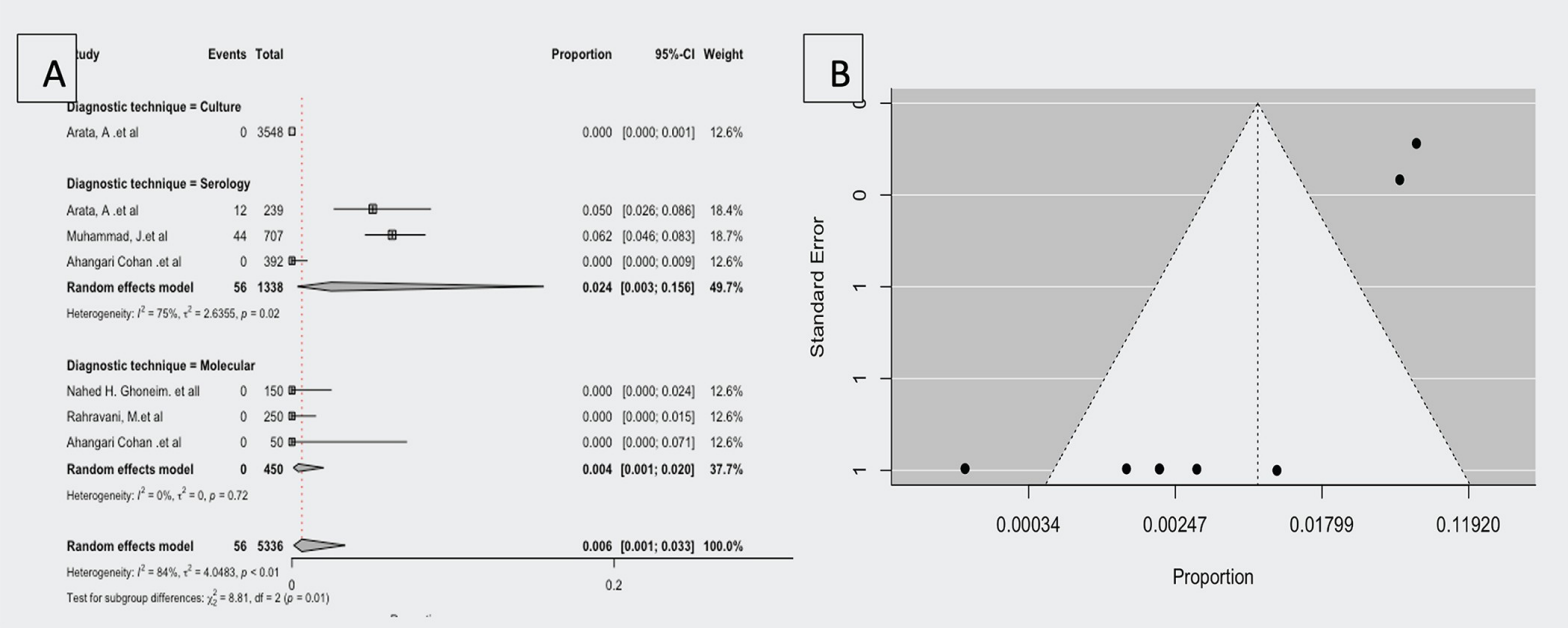
Forest plot showing the observed *F*. *tularensis* prevalence in ruminants and the estimate of the random-effects model, B; Funnel plot for a meta-analysis of studies of *F*. *tularensis* prevalence in ruminants.

The difference between the diagnostic technique subgroups was significant (Q (2) = 8.81, *p =* 0.012). The serology diagnosis technique has a higher proportion of the report μ = 0.024(95% CI, 0.003–0.156) than the molecular diagnosis technique (μ = 0.004; 95% CI, 0.001–0.020) and culture diagnosis technique, which is μ = 0.000 (95% CI, 0.000–0.001). Fig 7A shows a forest plot comparing the observed outcomes by diagnostic technique and estimates by random-effects model.

As shown in the studentized residuals, none of the studies had a value greater than 2.7344, indicating that this model contained no outliers. The overall proportion was 0.003 (95% CI, 0.001–0.009) when Cook’s distances removed three studies [1,2,6].

Fig 7B shows a funnel plot of the estimates. The regression test indicated funnel plot asymmetry (*p*<0.001), but the rank correlation test did not.

## Discussion

Tularemia is a disease that can be transmitted from animals and insects such as rodents, rabbits, hares, mosquitoes, ticks, and deer flies to humans. *F*. *tularensis* can survive in warm- and cold-blooded hosts such as mammals, arthropods, and freshwater protozoans (not firmly demonstrated), which shows its adaptability [38]. The bacteria found in the animal reservoir and the environment can increase the risk of infection in humans. This is attributed to the potential for animals in these regions to contaminate water sources with feces or carcasses, although the exact mechanisms of water contamination remain to be fully elucidated [39]. Drinking water contaminated by rodents and rabbits may be a source of contamination. Small water sources like ditches, lakes, ponds, and rivers are more vulnerable to contamination, but even large bodies of water can play a significant role in spreading infections [17]. The movement of infections is influenced by various factors such as human travel, animal migration, and climate [40]. Studies have shown that tularemia is widespread throughout Europe, Asia (type B), North America (type B and type A), and Russia. The widespread presence of *F*. *tularensis*, its diverse transmission modes, the geographical heterogeneity of its subspecies, and favorable environmental conditions all play a crucial role in the high prevalence of tularemia in Europe, Asia, North America, and Russia. Moreover, the application of multiple diagnostic approaches for tularemia has enhanced the availability of data on its prevalence in the region. Although tularemia is often misdiagnosed because of its rarity and lack of specificity in symptoms and clinical manifestations, it has recently been discovered that it can emerge as a disease in areas where it has not been reported for many years [41]. There has been a recent increase in tularemia cases in many Middle Eastern countries, owing to its re-emergence [7].

In our study the seroprevalence of tularemia in humans was 6.2%. Our research showed a slight variance between countries, and variations in prevalence over time were negligible in humans, without any link to quality scores. More than half of the research has been conducted over the last five years, with the majority in Iran, Egypt, and Jordan. The findings showed the hotspot of research on *F*. *tularensis* was from 2019 to 2020. In addition, seven epidemiological investigations on the prevalence of *F*. *tularensis* were conducted in Iran between 2013 and 2020. One study on the distribution of *F*. *tularensis* in people was conducted in Egypt in 2017, and another in Jordan in 2020. The seroprevalence of *F*. *tularensis* among humans was 5.5% (84 of 1317 samples) in Iran, whereas the seroprevalence was 9.3% (7 of 75samples) in Egypt and 7.7% (64 of 828 samples) in Jordan. Tularemia is presumably endemic in these countries. Further investigations are necessary to confirm this hypothesis. Tularemia is more common in some groups of people. Under the available research, at-risk groups for tularemia include laboratory employees, farmers, ranchers, hunters, veterinarians, nature conservation officers, butchers, and slaughterhouse workers [7].

The prevalence of *F*. *tularensis* in rodents was 2%. Rodents are natural hosts for *F*. *tularensis* and can carry live bacteria for an extended period while maintaining detectable antibody levels. Recently, there have been more reports of humans contracting tularemia from rodents [21]. However, information on the prevalence of tularemia in rodents in the Middle East is limited, with studies focusing mainly on Iran. Systematic epidemiologic studies are necessary to better understand the natural foci and the role of both wild and domestic animals in transmitting *F*. *tularensis* to humans.

Serological data are not equivalent to culture and PCR data for assessing disease prevalence in humans and animals. In environmental samples, molecular methods revealed a significantly higher prevalence (9.4%) compared to culture methods (0.5%). This finding highlights the inherent limitations of culture-based detection, as bacteria may be difficult to culture under certain environmental conditions. In ruminants, serological methods demonstrated the highest prevalence (2.4%) compared to culture (0.0%) and molecular methods (0.4%). This observation underscores the utility of serology in assessing historical exposure to *F*. *tularensis*, even when active infection is not present. *F*. *tularensis* is a biohazardous pathogen that necessitates specialized laboratories equipped for handling and analyzing this bacterium. Culture-based diagnosis of tularemia is relatively infrequent (≤ 10%) and often challenging due to the fastidious nature of the pathogen [42]. Serological tests, though not standardized, are widely used as the primary diagnostic method for tularemia. However, they are limited in terms of sensitivity and specificity. Combining multiple serological tests can increase diagnostic specificity while compromising sensitivity. Molecular methods, such as PCR, offer a more reliable and accurate approach to detecting tularemia, surpassing both serological and culture-based techniques [43].

Tularemia is a disease that is prevalent in certain parts of the Middle East, specifically in Iran, Egypt, and Jordan. Our study is the first to examine the epidemiology of tularemia in these regions in detail. We found that serology, molecular diagnostics, and arthropod surveillance are crucial for diagnosing and monitoring the disease. Additionally, we identified several groups of people who are at higher risk for contracting tularemia, including laboratory personnel, agricultural workers, hunters, veterinarians, conservation officers, meat handlers, and slaughterhouse employees. We recommend implementing targeted prevention strategies to protect these at-risk populations.

Our study on tularemia in the EMRO, focusing on Iran, Egypt, and Jordan, faces several limitations: The data primarily comes from a few countries, potentially not representing the region’s entire epidemiological picture. Most studies are recent, limiting long-term trend analysis. We largely relied on serological data, which may lack the sensitivity and specificity of culture and PCR methods, possibly leading to underestimations of prevalence. Additionally, there’s a scarcity of comprehensive studies on tularemia in both animals and environmental samples in the region, which hinders a complete understanding of the disease’s transmission dynamics. These factors underscore the need for broader, more systematic epidemiological research across the Middle East.

## Conclusion

According to the results of the present study, tularemia is an endemic but neglected disease in the WHO-EMRO region. However, most studies on tularemia are limited to a few countries in this region. Studies on tularemia in human populations, reservoirs, and vectors have been conducted in all countries in the WHO-EMRO region to obtain more detailed information about the epidemiology of tularemia in these regions.
